# Treatment outcomes in schizophrenia: qualitative study of the views of family carers

**DOI:** 10.1186/s12888-017-1418-8

**Published:** 2017-07-21

**Authors:** Joanne Lloyd, Helen Lloyd, Ray Fitzpatrick, Michele Peters

**Affiliations:** 1 School of Psychology, Sport and Exercise, Staffordshire University, Stoke on Trent, UK; 2 Peninsula Medical School, Plymouth University, Plymouth, Devon UK; 3 Nuffield Department of Population Health, University of Oxford, Old Road Campus, Oxford, OX3 7LF UK

**Keywords:** Schizophrenia, Treatment, Patient, Carer, Outcome

## Abstract

**Background:**

Schizophrenia is a complex, heterogeneous disorder, with highly variable treatment outcomes, and relatively little is known about what is important to patients. The aim of the study was to understand treatment outcomes informal carers perceive to be important to people with schizophrenia.

**Method:**

Qualitative interview study with 34 individuals and 8 couples who care for a person with schizophrenia/schizoaffective disorder. Interviews were transcribed verbatim and analysed by a thematic framework based approach.

**Results:**

Carers described well-recognised outcomes of importance, alongside more novel outcomes relating to: Safety (of the patient/others); insight (e.g. into non-reality of psychotic phenomena); respite from fear, distress or pain; socially acceptable behaviour; getting out of the house; attainment of life milestones; changes in personality and/or temperament; reduction of vulnerability to stress; and several aspects of physical health.

**Conclusions:**

These findings have the potential to inform the development of patient- or carer- focused outcome measures that take into account the full range of domains that carers feel are important for patients.

**Electronic supplementary material:**

The online version of this article (doi:10.1186/s12888-017-1418-8) contains supplementary material, which is available to authorized users.

## Background

Improving treatment outcomes and quality of life for people with long-term mental health conditions are key aims of health care policy [1, 2]. Schizophrenia is a particularly important target, being associated with poor quality of life [3] and individual and societal impacts [4–6], and requiring long-term treatment [7]. Antipsychotic medications can ameliorate some symptoms and improve quality of life [3, 8, 9], but individual responses vary [10, 11], and many discontinue medication due to poor efficacy or debilitating side effects [12, 13]. Treatment outcomes are often assessed by clinician ratings, and/or symptom scales [14], but patients and carers may prioritise different outcomes to clinicians [15–17], and controlling symptoms is not the only outcome of importance [14]. The recovery literature draws attention to the importance of recognising a broad array of outcome domains in schizophrenia treatment, highlighting the relevance of improved social and domestic functioning, alongside subjective wellbeing, optimism and empowerment (e.g. [18, 19]). Patients and relatives, in particular, refer to subjective wellbeing when defining ‘remission’, in contrast to traditional clinical definitions focused around reduced symptom scores [17]. People with schizophrenia value outcomes such as achieving life milestones, feeling safe, improved physical activity, employment, a positive sense of self and psychosocial outcomes [20]. Understanding the full range of treatment outcomes important to people with schizophrenia and their carers is key for ensuring that clinical practice, research and assessment are aligned with patient and carer priorities [4, 21].

While people with schizophrenia can give valid and reliable accounts of outcomes [22–24], symptoms can make it difficult to participate in research [25], and carers represent a valuable additional resource [15, 21, 26]. Furthermore, carers have the potential to influence treatment decisions [26], and experience, indirectly, the impact of outcomes. This study sought to explore the treatment outcomes that carers feel are important for people with schizophrenia. It used a framework informed by a thematic review of the existing literature on treatment outcomes of importance to patients and carers, and a consensus conference with professionals, carers and patients, and aimed to identify whether carers report any outcome domains that have not been emphasised in the current literature.

## Method

### Design of the study

A qualitative study using in-depth semi-structured interviews was conducted with self-identified ‘carers’ of a family member with a diagnosis of schizophrenia made at least 2 years previously. Ethical approval was obtained from NHS East of Scotland Research Ethics Service (EoSRES) REC 1 by proportionate review (Application Number 13/ES/0143). All participants gave written informed consent.

### Participants and recruitment

A total of 34 individuals and 8 couples were interviewed (i.e. 50 people in 42 interviews). While qualitative methodology papers tend to avoid prescribing hard guidelines for sample sizes for qualitative studies, 25–30 participants have been deemed an acceptable minimum by Dworkin [27] and this number is usually sufficient for reaching data saturation. An email circulated by charity ‘Rethink Mental Illness’ was responded to by 102 people who were screened via telephone to confirm that they were the carer of someone with a ≥ 2-year diagnosis of schizophrenia or schizoaffective disorder. Within this self-selecting convenience sample, participants were then recruited purposively to generate a relatively heterogeneous final sample, consisting of 38 females and 12 males, aged from 20s–80s (48% in their 60s, 26% in their 50s, and the remainder in their 20s, 40s, 70s or 80s), and coming from urban (e.g. Greater London) and rural (e.g. Wiltshire) locations. Thirty-seven were the mother of a person with schizophrenia, 10 were the father or stepfather, one the husband, one the wife, and one the sibling. Duration of illness of the patients discussed ranged from 2 to 20+ years, with a modal duration of 11–15 years (42%). The majority (*n* = 44) cared for someone with schizophrenia, and six cared for someone with schizoaffective disorder.

### Interviews

Most participants chose to be interviewed at home, but approximately 20% chose to come to the University. At the beginning of the interviews, carers re-confirmed that the patient had received a formal diagnosis of schizophrenia or schizoaffective disorder from a GP or psychiatrist, at least two years prior to the interview. Carers were then asked what they felt were important outcomes of treatment for the patient who they cared for: at present; at a time when the patient was particularly ill or unwell; at a time when they were more stable; and at a time when they were doing particularly well. Prompts were designed to encourage participants to discuss both directly-experienced outcomes, and important/desired but unattained outcomes. In addition, a series of prompts relating to key outcomes were compiled based on the conceptual review of the literature and feedback from a consensus conference, but were not in fact utilised in any of the interviews, as participants spontaneously discussed a broad array of outcomes of importance in response to the preliminary, general questions. After the initial 6 interviews, when it became apparent that participants identified multiple outcomes in response to the primary questions, without need for prompts, the researchers agreed that all future interviews in the study would proceed without prompts. Carers were encouraged to expand upon ideas that they themselves raised in relation to outcomes, rather than directed towards any specific topic. It was felt that this strengthened the data, as it reduced the potential for investigator bias. The topic guide, which was reviewed for tone and content prior to use by two carers and one person with schizophrenia, can be found in online Additional file 1. Interview duration ranged from 40 to 125 min (average, approx. 60 min).

### Analysis

Interviews were transcribed verbatim by a professional transcriber, and anonymised. Transcripts were analysed in NVivo 8 by JL, using a thematic, framework based approach [28]. This involved the creation of a preliminary framework based on a literature review and consensus conference. Transcripts were then analysed, with themes being coded into appropriate categories within that framework, wherever appropriate categories existed. Where themes did not fit well into an existing category, novel categories were created. Interviews were continued until no further novel categories emerged, by which point all categories had been spontaneously mentioned by several participants, and saturation was deemed to have been reached. Once all interviews had been coded, the categories were reviewed by the research team, to ensure that they were representative of all the statements coded within them. Where categories were ambiguous, e.g. contained material that could potentially be better conceptualised within different domains, or could be better represented by different titles, they were revised, and the material coded within them was re-coded in order to ensure that it was coded within the most appropriate category. A final framework that encompassed the original and the novel categories was then agreed amongst the researchers. All of the interviews were then re-coded, using the final framework. In this second iteration, the majority of the material was coded into the same categories as during the initial coding. However, this process was important to ensure that any statements that had originally been coded into categories within the preliminary framework, but in retrospect better-reflected a novel category that had been added to the final framework, were coded appropriately. RF and MP independently cross-checked the final categorisation by coding a random selection of 6 transcripts, and no disagreements emerged. Categorized data were summarized and synthesized, and the resultant categories (and associations between them) were interpreted in relation to the categories already identified within the literature and consensus conference. After the final coding, the number of interviews in which each category occurred was calculated.

## Results

### Overview

Outcomes of importance in schizophrenia reported by the carers included symptom related outcomes, quality of life, functional outcomes, personal recovery, physical health and lifestyle, and satisfaction with treatment. Table 1 lists these outcomes, and their sub-categories, and the proportion of interviews in which they occurred (using the conventions: ‘few’ for 2–10% (*n* = 1–4), ‘some’ for 12–24% (*n* = 5–10), ‘many’ for 25–50% (*n* = 11–21), and ‘most’ for >50% (*n* = 22–42)). It was not necessary for a participant to overtly state that an outcome had been experienced by the person they care for, in order to code their statement as an endorsement of that domain. While ‘endorsement’ of an outcome domain did, in some cases, take this form, any statement that either explicitly or implicitly indicated that a domain was relevant or important to that carer, was also coded within that domain. For example, where a carer identified that the person they cared for experienced ongoing difficulties with engaging in physical activity, or that they wished the person they cared for could have the energy to engage in physical activity, this was interpreted as the carer indicating that being able to engage in physical activity was an important outcome, and hence it was coded within the ‘physical activity’ category.

**Table 1:** Summary of treatment outcomes of importance (as identified by carers)

Symptom-related outcomes	
➢ Positive symptoms [50–53]^d^ ➢ Cognitive symptoms [50–53]^d^ ➢ Affective symptoms [50–53]^d^ ➢ *Safety* ^d^	➢ Negative symptoms [51–54]^d^ ➢ Stability (relapse/hospitalisation) [51, 55–57]^d^ ➢ Side-effects [21, 29, 58–60]^d^ ➢ *Fear/distress/pain* ^d^ ➢ *Insight* ^d^
Quality of life	
➢ Motivation and energy [61, 62]^d^ ➢ Physical functioning [61, 62]^a^ ➢ Life satisfaction [61, 62]^b^	➢ Emotional regulation/Self-control [61, 62]^c^ ➢ Stigma [63]^c^ ➢ Leading a normal life [50]^d^ */social acceptability* [61, 62]^d^
Functional outcomes	
➢ Independence [21, 53, 64]^d^ ➢ Role functioning/productivity^d^ (family/home/education/work/*pets* ^b^) [21, 29, 53, 64] ➢ *Life milestones* ^c^	➢ ADLs [15, 48]/Self-care [21, 53, 64]^d^ ➢ Social / relationships [21, 29, 53, 64]^d^ ➢ Leisure pursuits [53, 64] [34, 48]^d^ */getting out* ^d^
Personal recovery	
➢ Developing positive identity [65, 66]^d^ ➢ Finding meaning in life [65, 66]^c^ ➢ Accepting help/support from others [67]^d^ ➢ Learning about (& managing/monitoring) illness/treatment/services [68]^d^ ➢ Overcoming ‘stuckness’ [65, 66]^d^ ➢ *Vulnerability/sensitivity* ^d^	➢ Aiming for goals/‘new potentials’ [66, 67]^c^ ➢ Taking responsibility/decision-making [65]^d^ ➢ Developing hope/optimism [65, 67–69]^c^ ➢ Participating in life-enriching activities [66]^c^ ➢ Becoming more empowered [66–68]^c^ ➢ *Personality/temperament* ^d^
Physical health and lifestyle	
➢ *Exercise/physical activity* ^c^	➢ *Diet/weight* ^d^
➢ *Alcohol/drugs/smoking* ^c^	➢ *Routine* ^c^ ➢ Sleep^c^
Satisfaction with treatment	
➢ Satisfaction with medication [70]^c^	➢ Satisfaction with therapeutic relationship [71]^d^

Key: Number of interviews in which one or more of the carers referred to the outcome: ^a^few (*n* = 1–4), ^b^some (*n* = 5–10), ^c^many (*n* = 11–21), ^d^most (*n* ≥ 22). References cited indicate sources where the same treatment outcomes of importance have previously been identified. ADLs = activities of daily living

Outcomes presented in italics have not been described in previous studies but stem directly from the findings of this study

The categories in Table 1 were first identified through a literature review and consensus conference and subsequently adapted to include the newly identified and/or expanded categories from the interview data reported here. Standard font indicates categories which were pre-identified from the literature review (and replicated in the current study), and *italic* font indicates novel/ modified categories which emerged from the current study (which are illustrated by quotations in Tables 3 and 4, and discussed below). All categories in Table 1 were identified as relevant by at least some of the carers interviewed, and the majority were mentioned in >50% of the interviews.

### Symptom-related outcomes (Table 2)

Safety was mentioned in most interviews, and encompassed safety from dangerous behaviours prompted by psychosis (such as absconding/ putting oneself or others into risky situations); from health risks linked to negative symptoms (e.g. not eating, living in squalor); and from potential for deliberate self-harm related to affective symptoms.

**Table 2:** Quotations to illustrate symptom-related and quality of life related outcomes

Symptom-related outcomes	
*Safety (of self/others)*	
• ‘Her behaviour became so risky in a way, and it was so difficult with her at home.’ [C50]	
• ‘When he’s psychotic… he’s a danger to himself and he could be a danger to other people.’ [C26&27]	
• ‘You’re at risk the whole time and a bit more vulnerable.’ [C07]	
*Fear/distress/pain*	
• ‘It’s an effort and it’s almost a pain, it’s almost like a physical pain for him to stay normal.’ [C09]	
• ‘I know that he’s distressed by the psychosis. You know he’s seeing things that are really awful…	
• ‘If I look back, when medication was introduced [um] he probably was less distressed.’ [C24]	
• ‘I think the worst thing was that… why should he have so much suffering?’ [C13]	
*Insight*	
• ‘He’s doing very well at the moment although he’s in hospital. But the reason is that he has total insight… He knows what’s real… that has been the biggest progress.’ [C07]	
• ‘To recognise that it’s his illness that’s giving him his thoughts, that’s giving him his voices.’ [C09]	
• ‘She knows the difference between a paranoid thought and quote, a normal thought. So she knows when these thoughts are coming… and can begin to tackle them.’ [C22]	
• ‘He’s come to the realisation that he does need an anti-psychotic.’ [C11]	
Quality of life	
*Social acceptability*	
• ‘[Medication allows her] to behave appropriately and you know [um]… in family situations.’ [C04&05]	
• ‘Medication’s allowing him to be in the community and it’s allowing the community to be safe.’ [C12]	
• ‘Unusual behaviour. Is there anything that can be done about that?’ [C20]

*‘It's great for it to be diagnosed, to be put on your medication and you're safe’ [C41]*

The importance of reduction of, or relief from fear, distress and emotional (or even physical) pain was raised in most interviews, often closely related to positive symptoms, but at the level of their physical and emotional consequences.

*‘He was absolutely intimidated by his environment… he felt frightened and threatened’ [C25]*

Insight was also mentioned in most interviews, encompassing both recognition that current/prior psychotic phenomena are not real, and understanding that one has a long-term illness. It was described as a gatekeeper to many other treatment benefits, partly through its impact upon treatment adherence, and was important in helping people deal with residual psychotic phenomena.

*‘He can rationalise…although he hears the voices he has a sense of reality.’ [C40]*

Side-effects are not described in detail here as they are well reported within existing literature (e.g. [29]), but they were identified as important in the majority of interviews, and in addition to commonly-reported side effects (e.g. weight gain and fatigue), a few participants mentioned negative impact of medication on imagination and/or creativity, and concerns over toxicity of medication during pregnancy and breastfeeding.

### Quality of life (Table 2)

The concept of ‘social acceptability’ was raised in most interviews, i.e. behaving in a socially appropriate way and avoiding bizarre/unconventional behaviour. Many discussed the importance of treatment in helping patients avoid illegal behaviour (sometimes precipitated by symptoms).

*‘[When] he's not taking his medication, he occasionally offends people in the street’ [C25]*

### Functional outcomes (Table 3)

The domain of ‘life milestones’ was added to encompass many carers’ reports of the importance of reaching key life/developmental milestones, such as attaining qualifications, learning to drive, moving out of the caregiver’s home, or having a family.

*‘I think he missed out all his twenties and thirties so maybe catching up in some ways.’ [C03]*

Simply ‘getting out’ of the house was mentioned in most interviews, and was consequently added as a sub-category of ‘leisure pursuits’. This encompassed the importance of being well enough to leave the house, which was something many patients needed to achieve before the more ambitious step of engaging in structured leisure activities or even activities of daily living.

*‘The worst time that we've had was… when he was so unwell he didn’t go out the house for a year’ [C24]*

A novel sub-category of ‘pets’ was added within the ‘role functioning and productivity’ category, because the importance of being able to care for a pet was raised in some interviews.

### Personal recovery (Table 3)

The importance of ‘personality/temperament’ was raised in most interviews, and was often particularly valued by carers themselves. This encompassed emergence of aspects of the patient’s character, such as sense of humour, consideration, and thoughtfulness, and of a generally calmer temperament, more ‘like oneself’.

**Table 3:** Quotations to illustrate functional and personal recovery related outcomes

Functional outcomes	
*Life milestones*	
• ‘He said [um], “I’m grown up now Mum,” and he didn’t see me for a long time… as though he’s struck out to be an adult… but he wanted to start where he left off when he was a teenager.’ [C03]	
• ‘He wants to get married; he’d love to have children… you know he wants to have the life that everybody else has and he didn’t see himself not having that life and he’s like forty now and it’s like, you know, where have those twenty years gone when I should have been doing all this.’ [C32&33]	
• ‘Bear in mind she’s still, almost her development stopped when she was 17 as an adult.’ [C38]	
*Getting out*	
• ‘He’s been able to go out because before, he was frightened to go out not only because of what was out there, but because inside himself he couldn’t actually get out the door.’ [C06]	
• ‘For three years she didn’t really do anything, she just, for three years she was just at home… sleeping a lot and watching tele and not going out and generally not living.’ [C19]	
*Pets*	
• ‘He’d said… “I can’t manage myself let alone a dog”… it’s only recently that he said, “Oh yeah I think I’m up to having a dog,” so that’s a really good step forward.’ [C14&15]	
• ‘He loved the kitten but I thought he can look after the kitten… but he wouldn’t, he wouldn’t look after it, he just loved it. It’s a cat now and he still loves it but he still won’t look after it.’ [C51]	
Personal Recovery	
*Personality/temperament*	
• ‘He’s much easier when he’s on medication, he’s much more like himself and [um], oh god when he’s not on it I have to watch every word I say.’ [C12]	
• ‘I could see beneath his anxious anxiety and still his distress and everything, that there he was, my son that I knew whereas I’d lost sight of it completely before… I began to see the [1] that I knew before he became ill. He got his sense of humour back… I began to see his own personality coming back.’ [C09]	
• ‘Becoming more friendly, becoming more co-operative… more considerate, thoughtful.’ [C03]	
*Vulnerability/sensitivity*	
• ‘He’s very sensitive, very, very sensitive… he gets very stressed doesn’t he.’ [C14&15]	
• ‘Until the stress was sort of plonked upon him and then the medication wasn’t quite enough.’ [C23]	
• ‘It was times of stress and things…whether things went wrong then we’d get the paranoia then... he can’t take any stress that’s the thing.’ [C32&33]

*‘He reverted to his old self. You could reason with him, you could have a laugh with him’ [C46]*

The vast majority of carers also mentioned ‘vulnerability/sensitivity’ to all kinds of stress, in most cases as a residual difficulty that treatment failed to resolve, rather than a positive, attained outcome.

*‘Although he seems fairly even I don’t think it would take a huge amount to kick him over the edge.’ [C06]*

### Physical health and lifestyle (Table 4)

Exercise/physical activity and diet/weight were raised by the majority of carers, who sometimes described how treatment facilitated physical activity and healthy diet (by improving symptoms that create barriers), but also described how side-effects (such as alteration in appetite/metabolism, and fatigue) could act as barriers.

**Table 4:** Quotations to illustrate physical health and lifestyle related outcomes

Physical Health and Lifestyle
*Exercise/physical activity and diet/weight*
• ‘He was having plans. He said, “I need to go swimming, I want to get fit and I want to go swimming.” He wouldn’t go into the swimming pool because there are other people there.’ [C51]
• ‘The only pleasure he had in life in the end, after being institutionalised and losing all the interests that he’d got… was eating you know.. I’ll send out for a pizza, that’ll be nice and I’ll watch a DVD and that’s about as…and a couple cans of lager and that’s about as normal as things got for him really…’ [C46]
• ‘He can actually go down now and buy fresh fruit and vegetables… He couldn’t make decisions; [um] he didn’t know what food to eat… He didn’t have a fridge.’ [C25]
• ‘She got anorexic, she wasn’t eating at all; she wasn’t drinking because she thought the water was contaminated. Physically she was going downhill because she wasn’t eating or drinking.’ [C14&15]
*Alcohol/drugs/smoking*
• ‘He was self-medicating with alcohol. For quite a long while… he actually stopped drinking. And that helped a lot of things and it helped with the medication and generally with his mood.’ [C40]
• ‘Once they’ve become established, or on a working medication, they don’t need the cannabis. Maybe it’s they don’t need it, maybe that they’ve become well enough in order to have insight to know that it’s harmful to them.’ [C10]
• ‘She’s a confirmed smoker… It’s the least of our problems I think, you know.’ [C04&05]
*Routine*
• I feel a lot better now he’s at college because he has to get up, he has to go out, he’s got something to do. So that’s really good because it’s horrible to watch somebody doing nothing all day.’ [C21]
• ‘The only reason that [1]‘s working now… is because of this family… have made her do things and she’s living proof that if you get into a routine you can do things.’ [C38]
• ‘He has some physical problems with sleeping. His sleeping is a little erratic. He might not sleep very well at night but he catches up during the day. So his life balance is different to probably you or I. But he manages that in his way now.’ [C40]
• ‘They need a reason to get up, need a reason to get up in the morning’ [C28]

*‘On such a high dose… of a sedating medication. Motivation is just not there’. [C46]*

Many described the importance of outcomes related to drugs/alcohol/smoking, such as decreased reliance upon substances previously used to self-medicate positive or affective symptoms, or compensate for lack of social/functional activities.

*‘She was drinking herself to sleep, I think, mostly because she had recurrent nightmares, and day time nightmares’ [C50]*

Daily routine was mentioned in many interviews, in relation to sleep and waking, eating and self-care, and was described both as a factor that contributed to improving other outcomes, and as an outcome in itself.

## Discussion

### Principle findings

All the schizophrenia treatment outcomes identified in the literature review and consensus conference preceding the study (i.e. symptom-related outcomes; functional outcomes; personal recovery; quality of life; and satisfaction with treatment) were confirmed in these qualitative interviews, along with several novel sub-categories within existing domains and a novel category of physical health and lifestyle, thus giving a deeper understanding of outcomes in this condition. While a large proportion of the sample endorsed most of the themes, it should be noted that frequency information are indicative of the frequency of these domains within our sample, and cannot be extrapolated from to estimate the prevalence of these concerns in carers of persons with schizophrenia.

While the importance of physical activity for persons with schizophrenia is recognised within the literature [30], and low levels of physical activity have been demonstrated empirically to be associated with poorer outcomes in schizophrenia [31], its importance as a treatment *outcome* is not expressed in existing outcome measures. This highlights the need to consider physical activity as a potentially relevant outcome domain in its own right. Designing interventions for schizophrenia that include attention to physical health and lifestyle, could help improve outcomes for many patients.

Safety of the patient (and those around them), and reduction of their fear, distress or pain, were considered important by most carers, and it is easy to see why they would value these outcomes, relating to resolution of negative practical and emotional consequences of symptoms. While the importance of these outcomes may be intuitive, they are not explicitly represented in current outcome measures, and this study is novel in highlighting their particular salience. These outcomes could be described as ‘secondary’, in the sense that they could be logically expected to follow on from the more ‘primary’ outcome of amelioration of (particularly, positive) symptoms. However, it could also be argued that there are other means of reducing patients’ fear, distress, or pain, aside from by symptom resolution, and thus outcome measures could benefit from assessing the extent to which treatments help to reduce a patient’s experience of these negative states. This could help professionals to gain a fuller understanding of how a given treatment programme is impacting on the individual’s level of fear and distress.

Most carers also valued insight which they often reported to be associated with improved communication with the person with schizophrenia, and a return of their personality and/or of a more favourable, ‘normal’ temperament. This is consistent with findings that insight in schizophrenia is associated with social cognition [32], and lower scores on an aggression scale [33]. Carers also described insight’s importance for enabling patients to apply cognitive strategies to counter paranoid thoughts, delusions or hallucinations, consistent with the finding that insight can be predictive of prognosis [34]. Monitoring level of insight may be beneficial in order to inform decisions about *when* cognitive interventions may be more effective. Exploring the value of educating carers in ways to cope with poor insight in the person for whom they care, could be another important target for future work.

Within functional outcomes, many carers talked of ‘getting out’ (i.e. leaving the house), similar to the existing domain of engaging in leisure pursuits, but at a more preliminary level. Caring for pets, similarly, could be conceptualised as a specific form of role functioning/productivity. Where residual difficulties are considerable and/or recovery is particularly limited, less ‘ambitious’ functional outcomes such as these may be particularly relevant. This is consistent with the observation that traditional social functioning measures may not be relevant to people with severe disabilities related to schizophrenia [35], and with carers’ comments about reduced potential and lowering of expectations. From carers’ references to a range of key developmental/life events such as moving out of the family home, getting a job, learning to drive, and having a romantic relationship, we identified ‘reaching life milestones’ as an important and novel outcome. Because schizophrenia onset is typically during adolescence or early adulthood [36], before traditional milestones have been reached, it is logical that the *reaching* of milestones would for many be the goal, rather than the resumption of familial, domestic, occupational or educational roles and responsibilities. This highlights the fact that functional outcome measures in schizophrenia may need to take subtle levels of attainment into account, in order to accurately capture small gains.

Within the realm of ‘personal recovery’ many carers highlighted the importance of changes in personality and temperament, and several described the return of the person they used to know as *the* most important outcome; understandably so, considering that these are good outward indicators of wellness and ‘personal recovery’ and directly impact upon the patient-carer relationship. Indeed, temperament has been linked with functional outcomes and psychological health [37]. Also relating to personal recovery, many carers discussed patients’ vulnerability (to stress, and in general) and sensitivity, consistent with empirical findings of increased biological reactivity to stress in schizophrenia [38]. These were typically described as residual unresolved difficulties, and several carers reported that they limited patients’ attainment of functional outcomes and acted as precipitants of relapse, requiring careful monitoring. This could indicate a potential benefit to be found in involving carers, where appropriate, in helping patients to monitor level of stress, and react quickly to try and reduce its impact.

In the sub-category of ‘leading a normal life’, a number of carers spoke of the importance of treatment for helping patients to avoid socially unacceptable/antisocial/illegal behaviours, (often precipitated by positive symptoms), in order to reduce risk of arrest or sectioning, facilitate social interactions and minimise stigma – consistent with findings that socially unacceptable behaviour is strongly associated with stigma in schizophrenia [39].

Consistent with other studies, many carers expressed desire for greater monitoring of physical health [40]. Exercise/physical activity, diet, and weight were all salient concerns; again consistent with findings of elevated obesity [41] and low activity [42] in schizophrenia/severe mental illness. A wide range of contributing factors were cited by the carers, including medication side effects, positive, negative and affective symptoms, and eating replacing less attainable leisure pursuits. Several also described patients who used alcohol or drugs to self-medicate and/or compensate for a lack of alternative leisure outlets; consistent with reported motivations for substance use in schizophrenia [43]. Some carers did describe physical health benefits of treatment, e.g. where it reduced use of drugs or alcohol for self-medication, or reduced symptoms enough to allow patients to exercise or shop for healthy food. In relation to lifestyle more generally, several carers emphasised the importance of routine, as a desirable outcome and useful intervention for facilitating the attainment of other outcomes (consistent with a study where people with schizophrenia rated organization of time as a useful coping strategy [44]). The discovery that physical health is an important concern in schizophrenia is not novel, but this study does support the growing body of work emphasising the importance of incorporating physical health interventions into schizophrenia treatment programmes (e.g. [45]).

### Strengths and limitations

This study confirms the key treatment outcome categories found in the current literature, and contributes evidence of additional outcomes that carers feel are important for patients but are not apparently captured in current thinking about, and measurement of, schizophrenia outcomes. However, there are some possible biases in the sample. The majority of carers interviewed were parents of a person with schizophrenia, with a gender bias in the sample, such that around three quarters of participants were female. However, this is in line with the gender balance found in other convenience samples of carers of persons with schizophrenia [46], and reflects the fact that mothers are most frequently the primary carer in schizophrenia [47]. It is possible that spouses, siblings, or children (or those of a younger age in general) may have different perceptions of what the important outcomes are. Most participants were recruited via Rethink Mental Illness, which may have meant they were particularly well-informed about features of schizophrenia and issues around treatment. Finally, the patients discussed were typically quite advanced in chronicity (in most cases >10 years post-diagnosis). While carers were asked to discuss outcomes that they felt were important at different phases of illness, it is nevertheless possible that carers of patients more immediately post-diagnosis would report different outcomes. Future studies could benefit from exploring outcomes with younger carers with different relationships to the patient, from a range of backgrounds, and those caring for people more early post-diagnosis.

The outcomes carers identified as being important for patients may not be identical to the outcomes that patients themselves would identify. However, there is generally good agreement between the two [21], and as agents who potentially influence patients’ treatment decisions [16], and experience the consequences of the illness [48], carers’ views are important in their own right. Furthermore, we were able to gain insight into outcomes that might not otherwise have been represented, as most of the carers interviewed reported that the patients they were speaking about would have been unwilling/unable to participate (e.g. ‘he hates talking about it when he was really ill… he said, “It makes me feel so ill again” [C41]).

### Conclusions

The findings from this study contribute to our understanding of the full range of treatment outcomes that carers feel are important to people with schizophrenia, and could contribute to ensuring research, treatment planning and assessment are aligned with the needs and priorities of patients [4]. The breadth of information gleaned from these interviews with family carers indicates what an important resource this population represents. Furthermore, it is clear that informal carers typically bear a high burden of care in schizophrenia [49]. Working with carers to gain insights and coordinate interventions, where appropriate, could be a valuable way for professionals to develop person-centred approaches in schizophrenia. Outcomes of treatment should ideally be assessed with measures that both complement existing clinical scales and incorporate patient and carer priorities. The domains and more specific experience emphasised here should inform the further development of such patient- or carer- focused outcome measures in order to ensure more appropriate and complete evaluation of interventions.

## Additional file

Additional file 1: Lloyd et al., Treatment outcomes in schizophrenia: qualitative study of the views of family carers. Interview schedule. (DOCX 12 kb)
