# Supplementary material for: Treatment outcomes in schizophrenia: qualitative study of the views of family carers
Source: BMC Psychiatry. 2017 Jul 21;17:266. doi: 10.1186/s12888-017-1418-8 (PMC5521073; doi:10.1186/s12888-017-1418-8)
Supplement: Additional file 1: — Lloyd et al., Treatment outcomes in schizophrenia: qualitative study of the views of family carers. Interview schedule. (DOCX 12 kb) [file 12888_2017_1418_MOESM1_ESM.docx]

**Lloyd et al, Treatment outcomes in schizophrenia: qualitative study of the views of family carers**

# Interview schedule

1. Can you tell me a little about yourself? - 2.Can you tell me a bit about your current diagnosis? How long have you had this diagnosis? / Can you remember when you were first given this diagnosis?

3. Have you had any other diagnoses? If yes, can you tell me what they are/were?

4. Thinking about your health now... how do you feel that you are doing in general?

5. In what areas of your life do you feel you are doing well/not so well/having difficulties?

6. Currently, in which areas of your life /health do you feel it would be most important to see improvements? Are you currently receiving any form of treatment? What are your experiences of this treatment? What are the most important benefits or effects of the treatment(s)?

7. Can you tell me about when you have been unwell? Thinking back to this time period, what do you feel were the most important consequences or outcomes of the treatment you received?

8. Could we now talk about when you started to feel better/more stable? Thinking back to this time, what were the most important consequences or outcomes of the treatment you received?

9. Could we now talk about what happened when you started to feel a bit better/more stable? Thinking back to this time, what do you feel were the most important consequences or outcomes of the treatment you received?

10. Is there anything that we haven't covered that you feel is particularly important to mention in relation to our discussion?
